# Integration of AI and Machine Learning in Radiotherapy QA

**DOI:** 10.3389/frai.2020.577620

**Published:** 2020-09-29

**Authors:** Maria F. Chan, Alon Witztum, Gilmer Valdes

**Affiliations:** ^1^Department of Medical Physics, Memorial Sloan Kettering Cancer Center, New York, NY, United States; ^2^Department of Radiation Oncology, University of California, San Francisco, San Francisco, CA, United States

**Keywords:** artificial intelligence, machine learning, radiotherapy, quality assurance, IMRT, VMAT

## Abstract

The use of machine learning and other sophisticated models to aid in prediction and decision making has become widely popular across a breadth of disciplines. Within the greater diagnostic radiology, radiation oncology, and medical physics communities promising work is being performed in tissue classification and cancer staging, outcome prediction, automated segmentation, treatment planning, and quality assurance as well as other areas. In this article, machine learning approaches are explored, highlighting specific applications in machine and patient-specific quality assurance (QA). Machine learning can analyze multiple elements of a delivery system on its performance over time including the multileaf collimator (MLC), imaging system, mechanical and dosimetric parameters. Virtual Intensity-Modulated Radiation Therapy (IMRT) QA can predict passing rates using different measurement techniques, different treatment planning systems, and different treatment delivery machines across multiple institutions. Prediction of QA passing rates and other metrics can have profound implications on the current IMRT process. Here we cover general concepts of machine learning in dosimetry and various methods used in virtual IMRT QA, as well as their clinical applications.

## Introduction

Machine learning (ML) has the potential to revolutionize the field of radiation oncology in many processes and workflows to improve the quality and efficiency of patient care (Feng et al., 2018). The delivery of radiotherapy is complex and each step in the integrated process requires quality assurance (QA) to prevent errors and to ensure patients receive the prescribed treatment correctly. The recent research in machine learning efforts in the QA has produced a variety of proofs-of-concept, many with promising results (Kalet et al., 2020). In this article, we review the machine learning applications in radiotherapy QA.

The first question we seek to answer is why we want to integrate ML in radiotherapy QA. The term, machine learning, refers to the automated detection of meaningful patterns in data. In the past few years, it has become a major area of research and a common tool in many processes in radiotherapy (Feng et al., 2018). In this review paper, we will focus on machine learning applications to QA. As medical physicists, we perform an increasing number of QA tasks in our daily work, and prioritizing those that will help deliver the safest treatment is of paramount importance as stated in the American Association of Physicists in Medicine (AAPM) Task Group (TG) 100 (Huq et al., 2016). As such, learning from our QA data to choose those tasks that need early intervention is essential for our profession as more complex treatments are adopted. Currently, most of the data acquired during QA is utilized only as a one-time evaluation measurement but there is a lot of QA data available from which we can “learn” using machine learning methods and utilize past experience as knowledge.

This review will begin by introducing some general machine learning concepts for those who are not as familiar with this field. We will then combine these descriptions with explanations of their direct applications to QA. We also provide a non-exhaustive analysis of the literature on the applications of ML to QA data. This article hopes to demonstrate the power of machine learning and the advantages it offers to our QA programs.

### Artificial Intelligence and Machine Learning

Machine Learning maybe somewhat misleadingly referred as Artificial intelligence (AI), is already part of our everyday lives. The easiest way to explain the relationship of AI and ML is to visualize them as concentric circles with AI - the idea that came first, the largest; then ML - which blossomed later. In AI a general purpose algorithm that can reason about different problems is sought while in ML this idea is abandoned to search for a specific model that maps an input to an output using statistical learning techniques. Many classes of algorithms exist within ML that fit different functions, such as linear models like Lasso and Ridge regression (Hastie et al., 2009), Decision Trees (Luna et al., 2019); ensembles like Random Forrest and Gradient Boosting (Hastie et al., 2009), and Neural Networks (Rumelhart et al., 1986). All these algorithms are needed because one cannot guarantee a priori that an algorithm will be better than another in a random problem, a theorem knows as a no free lunch theorem (Wolpert, 1996). In practice, certain classes of algorithms work better than others in classes of problems. For instance, for the analysis of images, Convolutional Neural Network (CNN), a deep learning network, excels while for the analysis of tabular data Gradient Boosting has the lead. In the majority of problems. CNN uses convolution filters to extract general concepts that are later combined with other concepts that resemble how the visual cortex in animals works and puts emphasis in the local importance of each pixel (Le Cun and Bengio, 2002). Additionally, max pooling layers that take average of pixel and data augmentation techniques make it somewhat independent of translation and rotations of the images, all important part of their success. For the analysis of tabular data, however, this customization is not needed and an algorithm that is better at handling missing values, performs automatic feature selection, does not depend on monotonic transformations of the input variables and it is easy to train and regularize is more important. This is the case for Gradient Boosting (Friedman, 2001).

### Types of Learning

Machine learning algorithms use computational methods to “learn” information directly from data. There are two main types of learning: unsupervised learning and supervised learning. In unsupervised learning, the training data does not include label responses or desired outputs and the objective is to model the probability distribution of the given inputs. On the other hand, in supervised learning the training data does include labels or desired outputs and allows for the learning of a mapping between the input variables and the output (e.g., classification, regression, etc.).

#### Unsupervised Learning

Unsupervised learning is a type of machine learning algorithm used to draw inferences from datasets consisting of input data without labeled responses. The most common unsupervised learning method is cluster analysis, which is used for exploratory data analysis to find hidden patterns or grouping in data. The clusters are modeled using a measure of similarity (MathWorks.com, 2020). Li et al. utilized unsupervised learning tools of K-means and hierarchical clustering algorithms to analyze patients' breathing curves extracted from 4D radiotherapy data (Li et al., 2017). The authors classified patients' breathing patterns into sub-groups, such as perfect, regular, and irregular breathers. The breathing signals and frequency spectrum were extracted from 341 real-time position management (RPM) datasets. Correlation plots of 6 features (frequency, amplitude, standard deviation of amplitude, spread of frequency spectrum) were chosen for the clustering task. Two clustering algorithms were used by the authors: hierarchical clustering and k-means. Hierarchical clustering generates more consistent results than k-means but requires a more (and usually prohibiting) training time than k-means (Li et al., 2017). This could lead to inefficiency in large datasets. K-means is extremely sensitive to cluster center initialization; therefore, some degrees of prior knowledge about the data is required for its effective usage. We will also demonstrate that the same RPM data could be used for both unsupervised and supervised learning to achieve different goals, although this topic might not be directly related to radiotherapy QA.

#### Supervised Learning

Supervised learning is the machine learning task of learning a function that maps an input to an output based on example input-output pairs (Russell and Norvig, 2010). A supervised learning algorithm takes a known set of input data and responses (output) to learn the regression/classification model. A learning algorithm is then used to train a model and generate a prediction for the response to new data or the test dataset. When statistical learning algorithms are used (e.g., Random Forest, Gradient Boosting, Decision Trees) features that are expected to describe the output need to be defined and calculated (Shobha and Rangaswamy, 2018). Therefore, for each observation features are extracted and associated with the label sought to be predicted. We can then use these features and output to learn a mapping from one to the other using ML algorithms. Thus, when a new IMRT plan is generated, the same features can be extracted to be used in the trained predictive model to show the expected label such as pass/fail (classification) or passing rate (regression). This is the approach first proposed in Virtual IMRT QA (Valdes et al., 2016) and further validated (Valdes et al., 2017).

Supervised learning was also used with the same RPM data described in section Unsupervised Learning above (Lin et al., 2019). With over 1,700 RPM data from 3 institutions, a Long short-term memory (LSTM) model was built by Lin et al. to predict different types of patients' respiratory motions in real-time (Lin et al., 2019). LSTM is a recurrent neural network (RNN) recently designed to alleviate the issues with vanishing gradients seen in earlier RNN. LSTM is specifically useful for the analysis of sequence data like text or this RPM data (Lin et al., 2019). In this study, the authors used a sliding window technique to partition the RPM data into the input and supervised output. This study demonstrates the potential of using deep learning models in respiratory signal prediction and incorporating the motion into treatments. This example, though slightly removed from radiotherapy QA, is chosen to emphasize the fact that applying different learning algorithms on the same dataset could serve different purposes.

#### Semi-supervised Learning

Semi-supervised learning falls between unsupervised learning and supervised learning. In semi-supervised learning, part of the training data does not contain a label. However, by leveraging the correlation structure of the input variables, a model that explains the label portion is obtained. Naqa et al. performed a multi-institutional study with data from eight Linacs and seven institutions (El Naqa et al., 2019). The authors investigated the use of machine learning methods for the automation of machine QA. A total of 119 EPID (electronic portal imaging device) images of a special QA phantom were fed into the support vector data description (SVDD) clustering algorithm (unsupervised learning). QA test data was first mapped to a higher dimensional space to identify the minimal enclosing sphere. This sphere was then mapped back to the input space to detect outliers. The separate clusters generated were further used to evaluate the tolerance boundaries and limits as indicated in the AAPM TG-142. The prediction tests included gantry sag, radiation field shift, and multileaf collimator (MLC) offset data. This study demonstrated that machine learning methods with SVDD clustering are promising for developing automated QA tools.

### Validation of ML Models

In machine learning, model validation is referred to as the process where a trained model is evaluated with a testing data set. The test data set should be a separate portion of the same data set from which the training set is derived. The main purpose of using the testing data set is to validate the generalizability of a trained model (Alpaydin, 2010). Validation of a predictive model is an essential part of the model building process, and is used to assess the quality of a model. When conducting a machine learning study, commonly used validation methods include: (1) using different machine learning algorithms on the same data to compare the results, (2) using cross-validation to obtain an error estimation on out of sample data, (3) using a hold-out sample for testing, (4) comparing with other well-established models that are not necessary machine-learning models, (5) validating using a sample not from the training period but acquired at a later time, (6) validating using a sample that is selected from a different population than that used to build the model (e.g., different clinic). Model validation is usually carried out after model training to find the optimal model with the best performance. The two most popular types of validation methods used in predictive models of radiotherapy QA are splitting training/test/holdout datasets and k-fold cross-validation. There are multiple ways to split the data. One method is to split the data pool into roughly 70% used for training the model and 30% for testing the model, and another method splits the data into three with, for example, 60% for training, 30% for testing, and the remaining 10% for holdout. Validating on the holdout set is done to check if the model suffers from overfitting due to optimization of the model hyperparameters. Instead of the data splitting as described above, k-fold cross-validation splits the data into k folds, then trains the data on k-1 folds and tests on the remaining fold to evaluate the model (Alpaydin, 2010; Russell and Norvig, 2010). The procedure is repeated k times with a different group of observations treated as a validation set each time. The most frequently used in radiotherapy QA applications is either 5- or 10-fold cross-validation. The model accuracy can be evaluated using a variety of metrics including, but not limited to, the mean squared error (MSE), root mean square error (RMSE), mean absolute error (MAE), receiver operating characteristic (ROC), correlation coefficient, regression plot, residual error histogram, sensitivity and specificity.

## Machine Learning Applications in Machine QA

In this section, we will focus on the general applications of ML to Linac QA before discussing IMRT QA. There have been many studies of machine learning applications in Linac QA including prediction of machine dosimetry as well as discrepancies of MLC positioning and their impact on the actual dose delivery.

### ML Model Built From Dosimetric QA or Beam Data

Another application, Li and Chan developed a model to predict the performance of Linac over time (Li and Chan, 2017). The study applied Artificial Neural Networks (ANNs) time-series prediction modeling to the longitudinal data of 5-years of daily Linac QA. A set of one hidden layer, six hidden neurons, and two input delays were chosen after a trial-and-error process to form the network architecture. The predictive model was compared with a well-established model, autoregressive integrated moving average (ARIMA). The ANN time-series mode was found to be more accurate than the ARIMA techniques to predict the Linac beam symmetry accurately (Li and Chan, 2017). Zhao et al. (in press) utilized 43 sets of commissioning and annual QA beam data from water tank measurements to build a machine learning model that could predict the percent depth doses (PDD) and profiles of other field sizes such as 4 × 4 cm^2^, 30 × 30 cm^2^ accurately within 1% accuracy with 10 × 10 cm^2^ data input. This application would potentially streamline the data acquisition for the entire commissioning process in TPS as well as optimize periodic QA of Linacs to a minimum set of measurements.

### ML Model Built From Delivery Log Files

Carlson et al. were the first to use machine learning techniques to train models to predict these discrepancies (Carlson et al., 2016). Predictive leaf motion parameters such as leaf position and speed were calculated for the models. Differences in positions between synchronized DICOM-RT files and Dynalog files from 74 VMAT plans were used as a target response for training the models. Three machine learning algorithms were used—linear regression, random forest, and a cubist model. They found that the cubist model outperformed all other models in terms of accuracy to predict MLC position errors. The objective of these predictions was to incorporate them into the TPS and provide clinicians with a more realistic view of the dose distribution as it will truly be delivered to the patient. Osman et al. (2020) collected 400 delivery log files and trained a model with feed-forward ANN architecture mapping the input parameters with the output to predict the MLC leaf positional deviations with a train/test split of 70 and 30%. The ANN model achieved a maximum MSE of 0.0001 mm^2^ in predicting the leaf positions for each leaf in the test data. The results of the study could be extended to utilizing this information in the dose calculation/optimization algorithm. Chuang et al. developed a machine learning model using prior trajectory log files generated from 116 IMRT and 125 VMAT plans to predict the MLC discrepancies during delivery and provide feedback of dosimetry (Chuang et al., in press). A workflow was developed to extract discrepancies and mechanical parameters from trajectory logs and use the proposed machine learning algorithm to predict discrepancy. The authors used multiple machine learning models including linear regression, decision tree, and ensemble methods.

### ML Model Built From Proton Fields

Sun et al. used 1,754 proton fields with various range and modulation width combinations to train an output factor (OF) model in three different algorithms (Random Forest, XGBoost, and Cubist) with a train/test split of 81 and 19% (Sun et al., 2018). They found that the Cubist—based solution outperformed all other models with a mean absolute discrepancy of 0.62% and maximum discrepancy of 3.17% between the measured and predicted OF. They concluded that machine learning methods can be used for a sanity check of output measurements and has the potential to eliminate time-consuming patient-specific measurements. Similarly, Grewal et al. utilized 4,231 QA measurements with a train/test split of 90 and 10% to build models to predict OF and MU for uniform scanning proton beams with two learning algorithms—Gaussian process regression and shallow neural network (Grewal et al., 2020). They found that the prediction accuracy of machine and deep learning algorithms is higher than the empirical model currently used in the clinic. They have used these models in the clinic as a secondary check of MU or OF.

Table 1 lists the studies on radiotherapy machine QA using machine learning techniques. All of these studies showed that machine learning techniques can give physicists insights into past QA data and to predict potential machine failures. This would alert physicists to take proactive actions and make informed decisions.

**Table 1:** Summary of studies on machine QA using machine learning techniques in a chronological order.

**References**	**QA Source**	**Data Source**	**ML Model**	**Task**
Carlson et al. (2016)	DICOM_RT, Dynalog files	74 VMAT plans	Regression, Random Forest, Cubist	MLC Position Errors Detection
Li and Chan (2017)	Daily QA Device	5-year Daily QA Data	ANN Time-Series, ARIMA Models	Symmetry Prediction
Sun et al. (2018)	Ion Chamber	1,754 Proton Fields	Random Forrest, XGBoost, Cubist	Output for Compact Proton Machine
El Naqa et al. (2019)	EPID	119 Images from 8 Linacs	Support Vector Data Description, Clustering	Gantry Sag, Radiation Field Shift, MLC Offset
Grewal et al. (2020)	Ion Chamber	4,231 Proton Fields	Gaussian Processes, Shallow NN	Output and Patient QA Proton Machine
Osman et al. (2020)	log files	400 machine delivery log files	ANN	MLC Discrepancies during Delivery & Feedback
Chuang et al. (in press)	Trajectory log files	116 IMRT plans, 125 VMAT plans	Boosted Tree Outperformed LR	MLC Discrepancies during Delivery & Feedback
Zhao et al. (in press)	Water Tank Measurement	43 Truebeam PDD, Profiles	Multivariate Regression (Ridge)	Modeling of Beam Data Linac Commissioning

## Machine Learning Applications in IMRT/VMAT QA

This section will now focus on describing the applications of Machine Learning to IMRT QA. Features can be extracted from each IMRT plan and compute multiple complexity metrics associated with passing rates. These features can be used to build a model that can predict the passing rate for any new IMRT plan.

### ML Applied to IMRT QA

#### Early ML Models

Valdes et al. developed the first virtual IMRT QA using a Poisson regression machine learning model to predict passing rates (Valdes et al., 2016). The initial dataset contained 498 clinical IMRT plans from the University of Pennsylvania, with QA results from a MapCHECK (Sun Nuclear Corporation, Melbourne, FL) QA device. An additional dataset was obtained containing 203 clinical IMRT beams also planned from Eclipse (Varian Medical Systems, Palo Alto, CA) but QA results were obtained using portal dosimetry. The plans from the University of Pennsylvania were used to identify 78 important features. Additionally, 10 further features were added to take into account the specific characteristics of portal dosimetry (Valdes et al., 2017). All parameters of each IMRT beam were automatically extracted from Eclipse with SQL queries and scripts were written to read the MLC positions and collimation rotation from the files. Matlab (The MathWorks Inc., Natick, MA) functions were developed to calculate the features for each beam. For MapCHECK, the important features extracted included the fraction of area delivered outside a circle with a 20 cm radius (to capture symmetry disagreements), duty cycle, the fraction of opposed MLCs with an aperture smaller than 5 mm (to quantify the effects of rounded leaves in the MLC), etc. For portal dosimetry, the important features included the CIAO (Complete Irradiated Area Outline) area, the fraction of MLC leaves with gaps smaller than 20 or 5 mm, the fraction of area receiving <50% of the total calibrated MUs, etc. (Valdes et al., 2017).

A machine learning algorithm was trained to learn the relationship between the plan characteristics and the passing rates. There are 80 complexity metrics being used in the calculation in the initial modeling with Penn data using the MapCHECK QA data. A learning curve for the initial model was established to show that around 200 composite plans are needed to adequately train the model. A strong correlation between the MapCHECK measurement and virtual IMRT predicted passing rates for data that the algorithm had not seen was obtained. All predictions of passing rates were within ±3% error.

For the portal dosimetry model, a learning curve was also performed to estimate the number of IMRT fields needed, and it was shown that close to 100 individual IMRT fields are sufficient to build a reliable predictive model. In total there were 90 continuous variables used for the virtual IMRT QA model which predicted EPID panel passing rates. The authors presented the residual errors of the passing rates prediction for the two institutions (the University of Pennsylvania and Memorial Sloan Kettering Cancer Center). Although the passing rates are site-dependent, different models were not built for each site because, conditional on the plan characteristics, this dependency disappears.

In order to implement virtual IMRT QA in a clinic the following workflow should be followed: (1) collect or access IMRT QA data, (2) extract all the parameters of the IMRT fields from plan files, (3) extract the features for the calculation of all complexity metrics affecting the passing rates, (4) use a machine learning algorithm to build a virtual IMRT QA model. During this process, we identify the most impactful features that affect the passing rate.

#### Deep Learning Models

The process described in the previous section Early ML Models requires carefully designing features that describe the correlation between plan characteristics and passing rates. Using an algorithm capable of designing their own features, Dr. Valdes and his group compared a Deep Neural Network against their own Poisson regression model using the same patient QA data previously described (Interian et al., 2018). The input to the CNN, a special type of neural network designed to analyze images, was the fluence map for each plan without the need of expert designed features. The models were trained to predict IMRT QA gamma passing rates using TensorFlow and Keras. The authors concluded that CNNs with transfer learning can predict IMRT QA passing rates by automatically designing features from the fluence maps without human expert supervision. The predictions from the CNNs were comparable to the virtual IMRT QA system described above which was carefully designed by physicist experts.

Tomori et al. built a prediction model for gamma evaluation of IMRT QA based on deep learning (Tomori et al., 2018) using sixty IMRT QA plans. Fifteen-layer CNN were developed to learn the planar dose distributions from a QA phantom. The gamma passing rate was measured using EBT3 film. The input training data also included the volume of PTV, rectum, and overlapping region, and the monitor unit for each field. The network produced predicted gamma passing rates at four criteria: 2%/2 mm, 3%/2 mm, 2%/3 mm, and 3%/3 mm. Five-fold cross-validation was applied to validate the performance. A linear relationship was found between the measured and predicted values for all criteria. These results also suggested that deep learning methods may provide a useful prediction model for gamma evaluation of patient-specific QA.

Lam et al. applied 3 tree-based machine learning algorithms (AdaBoost, Random Forest, and XGBoost) to train the models and predict gamma passing rates using a total of 1,497 IMRT beams delivered with portal dosiemtry (Lam et al., 2019). They reported that both AdaBoost and Random Forest had 98 ± 3% of predictions within 3% of the measured 2%/2 mm gamma passing rates with a maximum error <4% and a MAE <1%. XGBoost showed a slightly worse prediction accuracy with 95% of the predictions <3% of the measured gamma passing rates and a maximum error of 4.5%. The three models identified the same nine features in the top 10 most important ones that are related to plan complexity and maximum aperture displacement from the central axis or the maximum jaw size in a beam. Their results demonstrated that portal dosimetry IMRT QA gamma passing rates can be accurately predicted using tree-based ensemble learning models.

Nyflot et al. investigated a deep learning approach to classify potential treatment delivery errors and predict QA results using image and texture features from 186 EPID images (Nyflot et al., 2019). Three sets of planar doses were exported from each QA plan corresponding to (a) the error-free case, (b) a random MLC error case, and (c) a systematic MLC error case. Each plan was delivered to an EPID panel and gamma analysis was performed using the EPID dosimetry software. Two radiomic approaches (image and texture features) were used. The resulting metrics from both approaches were used as input into four machine learning classifiers in order to determine whether images contained the introduced errors. After training, a single extractor is used as a feature extractor for classification. The performance of the deep learning network was superior to the texture features approach, and both radiomic approaches were better than using gamma passing rates in order to predict the clinically relevant errors.

### ML Applied to VMAT QA

ML applications have been extended to volumetric modulated arc therapy (VMAT) QA. Granville et al. built a ML model with 1620 VMAT plans (Elekta) to predict the results of VMAT QA measurements using not only treatment plan characteristics but also Linac performance metrics (Granville et al., 2019). They trained a linear Support Vector Classifier (SVC) to classify the results of VMAT QA. The outputs in this model were simple classes representing the median dose difference (±1%) between measured and expected dose distributions rather than passing rates. In the model development phase, a recursive feature elimination (RFE) cross-validation technique was used to eliminate unimportant features. Of the ten features found to be most predictive of VMAT QA measurement results, half were derived from treatment plan characteristics and a half from Linac QA metrics. Such a model has the potential to provide more timely failure detection for patient-specific QA. Ono et al. utilized 600 VMAT plans and their corresponding ArcCHECK measurements to build prediction models using three machine learning algorithms—regression tree analysis, multiple regression analysis, and neural network. They found that the neural networks model achieved slightly better results among the 3 models in terms of prediction error (Ono et al., 2019).

Li et al. investigated the impact of delivery characteristics on the dose accuracy of VMAT (Li et al., 2019a). Ten metrics reflecting VMAT delivery characteristics were extracted from 344 QA plans. The study found that leaf speed is the most important factor affecting the accuracy of gynecologic, rectal, and head and neck plans, while the field complexity, small aperture score, and MU are the most important factors influencing the accuracy of prostate plans. Li et al. also studied the accuracy of prediction using machine learning for VMAT QA (Li et al., 2019b). The authors presented the workflows for two prediction models; the classic Poisson regression model, and the newly constructed Random Forest classification model. To test the prediction accuracy, 255 VMAT plans (Varian) with 10-fold cross-validation were used to explore the model performance under different gamma criteria and action limits. In clinical validation, independent 48 VMAT plans without cross-validation were used to further validate the reliability of models. The authors also showed the absolute prediction error with both technical and clinical validations. The prediction accuracy was greatly affected by the absolute value of the measured gamma passing rates and gamma criteria. The regression model was able to accurately predict those passing rates for the majority VMAT plans, but the classification model had a much better sensitivity to accurately detect failed QA plans. Later the same group further improved their prediction model using autoencoder based classification-regression (ACLR) to generate gamma passing rates predictions for three different gamma criteria from 54 complexity metrics as input (Wang et al., in Press). With an additional 150 VMAT plans available for clinical validation to evaluate the generalized performance of the model, the group reported that such a hybrid model significantly improved prediction accuracy over their early model, Poisson Lasso regression.

Wall and Fontenot used 500 VMAT and MapCHECK2 data to build predictive models using four different machine learning algorithms and then compared their performance (Wall and Fontenot, 2020). They found that the SVM model, trained using the 100 most important features selected using the linear regression method, gave the lowest cross-validated testing MAE of 3.75% as compared to linear models, tree-based models, and neural networks. More recently, Hirashima et al. (2020) used Gradient Boosting, the most accurate algorithm up to date for the analysis of tabular data, to create a model to predict ArcCHECK measurements using plan complexity and dosiomic features extracted from 1,255 VMAT plans, also showing the validity of virtual VMAT QA.

Table 2 lists the studies on virtual IMRT/VMAT QA. In short, there have been multiple studies that all find similar conclusions independent of the brand of Linac, TPS, and QA tool used: QA results can be predicted accurately using machine learning.

**Table 2:** Summary of studies on patient-specific QA using machine learning techniques.

**Group**	**TPS/Delivery**	**QA Source**	**Data Source**	**ML Model**	**Research Highlight**
Valdes et al. (2016)	Eclipse/Varian	MapCHECK2	498 IMRT Plans	Poisson Regression	Founding Paper
Valdes et al. (2017)	Eclipse/Varian	Portal Dosimetry	203 IMRT Beams	Poisson Regression	Multi-sites Validation
Interian et al. (2018)	Eclipse/Varian	MapCHECK2	498 IMRT Plans	Convolutional Neural Network	Fluence Maps as Input
Tomori et al. (2018)	iPlan/Varian	EBT3 film	60 IMRT Plans	Convolutional Neural Network	Planar Dose, Volumes, MU
Lam et al. (2019)	Eclipse/Varian	Portal Dosimetry	1,497 IMRT Beams	AdaBoost, Random Forest, XGBoost	Tree-based High Accuracy
Nyflot et al. (2019)	Pinnacle/Elekta	EPID	186 IMRT Beams	Convolutional Neural Network	Image, Texture Features
Granville et al. (2019)	Monaco/Elekta	Delta4	1,620 VMAT Beams	Support Vector Classifier	1st VMAT & w/ QC Metrics
Ono et al. (2019)	RayStation, Eclipse/Vero, Varian	ArcCHECK	600 VMAT Plans	Regression Tree, Multiple Regression, Neural Network	ML Models Comparison
Li et al. (2019b)	Eclipse/Varian	MatriXX	255 VMAT Beams	Poisson Lasso & Random Forest	Specificity & Sensitivity
Wang et al. (in Press)	Eclipse/Varian	MatriXX	576 VMAT Beams	Hybrid Model ACLR	High Prediction Accuracy
Wall and Fontenot (2020)	Pinnacle/Elekta	MapCHECK2	500 VMAT Plans	Linear Regression, SVM, Tree-based, ANN	ML Models Comparison
Hirashima et al. (2020)	RayStation, Eclipse/ Vero, Varian	ArcCHECK	1,255 VMAT Plans	Hybrid Model XGBoost	Plan Complexity & Dosiomics

## Summary and Future Directions

Since the early ML models applied to machine and patient-specific QA were reported in early 2016, a significant improvements have been seen in more recent models as machine learning techniques in radiotherapy QA matured. The models grew from simple Poisson regressions to deep learning classification models, and then to complex hybrid models which improved prediction accuracy. Therefore, it is expected that future ML models built on the foundation of existing knowledge can continue to be refined. With deep learning models, there is a greater potential to make QA processes more efficient and effective in clinical settings. In the meantime, it is very important to fully understand the limitations of virtual QA. Kalet et al. has highlighted some of the unique challenges of ML applications in radiotherapy QA including data quality, model adaptability, and model limitations (Kalet et al., 2020). Data quality is by far the most basic and essential requirement for building an accurate prediction model. Not only can incomplete data, such as small sample size, lead to wrong conclusions, but “true” QA data from detectors, especially for extremely small/large field size or large low dose regions, can also lead to imperfect prediction models due to detector system limitations (Valdes et al., 2017). Multi-institutional validation is often helpful to validate and generalize the ML models. In addition to the challenges of data integrity, Kearney et al. raised awareness of some persistent misuse of deep learning in the field (Kearney et al., 2018).

To date, many applications of ML to radiotherapy QA have focused on predicting machine performance and IMRT/VMAT QA results. Fully understanding and dissecting all factors that govern delivery accuracy is extremely important for clinical physicists to be able to implement a risk-based program as suggested in the AAPM TG-100 report. Further developments could lead to QA predictions being included in the treatment planning optimizer so that all QA could pass. We could also know ahead of time that we need to run a clinically-relevant QA on those plans with the lowest expected passing rates. It is clear that prediction of QA results could have profound implications on the current radiotherapy process. Before implementing in-house or commercial ML models to perform sanity check, second check, and automated or virtual QA in any clinical setting, we should carefully assess and address the limitations of both data and ML models.

## Author's Note

The materials were presented in a SAM Therapy Educational Course at the 61st AAPM Annual Meeting in San Antonio, TX, in July 2019.

## Author Contributions

MC, AW, and GV have contributed to writing this review article. All authors contributed to the article and approved the submitted version.

## Conflict of Interest

MC has no commercial or financial interests to declare. AW and GV report an ownership stake in Foretell Med LLC, which is developing machine learning models in medicine. However, this work was done before and outside of that company.
